# Against the Stream: lowering the age of sexual consent

**DOI:** 10.1192/bjb.2017.26

**Published:** 2018-08

**Authors:** Philip Graham

**Affiliations:** 1 University College London, UK.

## Abstract

Lowering the legal age of sexual consent would decriminalise a large number of ‘underage’ young people engaging in sexual intercourse. The arguments against such a change in the law are summarised and shown to lack validity.

**Declaration of interest:**

None.

The proposal that the age of consent should be lowered is not just ‘against the stream’. It is regarded by many as a preposterous idea. When, in 2013, the then President of the Faculty of Public Health, Professor John Ashton, made the proposal that the age of consent should be reduced from 16 years to 15 or even 14 years, it was immediately rejected by both government and opposition spokesmen.^1^ Indeed, David Cameron, when Prime Minister referred to the proposal as ‘offensive’.

## The present legal situation

It is the case that there are many cogent arguments against such a move. It will be claimed here that none of these is valid. Further, it will be proposed not only that there would be specific advantages to changing the law in this way, but that the principles on which this proposal is based have implications for other ways in which the rights of young people are inappropriately curtailed.

The existing laws in all the jurisdictions of the UK state^2^ that the age of consent for any form of sexual activity is 16 years for both men and women. The age of consent is the same regardless of the gender or sexual orientation of a person and whether the sexual activity is between people of the same or different gender.

It is an offence for anyone to have any sexual activity with a person under the age of 16. However, Home Office guidance is clear that there is no intention to prosecute teenagers under the age of 16 where both mutually agree and where they are of a similar age.^3^ Further, it is an offence for a person aged 18 or over to have any sexual activity with a person under the age of 18 if the older person holds a position of trust (for example, a teacher or social worker) as such sexual activity is an abuse of the position of trust.

There is wide variation between countries in the age of sexual consent. In Europe, all countries have minimum age limits for sexual relations. Nowhere is this age set lower than 14 years.^4^ In Europe, consensual relations with 14-year-olds are legal in half of the jurisdictions, and with 15-year-olds in three-quarters.^5^ This places the UK among the quarter with the most restrictive legislation. There is no evidence that the legal minimum age of sexual consent in a country is in any way correlated with the sexual behaviour of young people.

Various principles have been adduced which should govern the legal position of minors.^6^ Although it is often implied that children should not be regarded as such, Waites^6^ (p. 218) suggests that children are indeed citizens who, like adult citizens, have a right to protection as well as a right to freedom of activity. He argues that sexual behaviour below the age of 14 should be criminal, and that there is a role for legal prohibitions for the collective good which goes beyond preventing harm in individual cases (pp. 220–241).

## The arguments against

The following arguments have been used against the proposal to lower the age of sexual consent. These are followed by counter-arguments.

(a) A change in the law would result in more younger children becoming inappropriately engaged in sexual activity. There is a lack of evidence this is the case and, indeed, much evidence suggesting that the existing law has no effect on the sexual behaviour of young people. Information collected between 2010 and 2012 suggests that 31% of British males and 29% of British females had full sexual intercourse before the age of 16 years. Fifty years previously, this had been the case for 15% of males and 4% of females.^7^ There had been no change in the law in relation to heterosexual intercourse in the interim. A study of the reasons for sexual abstinence in American school students revealed that the law was not cited as a reason for abstaining from sexual activity.^8^
(b) The existing law gives young people, especially girls, who do not want to engage in sexual activity a powerful reason for refusing to consent. Although this reason is often cited, there is not even anecdotal evidence to suggest it is valid. It is indeed difficult to imagine a girl saying to her boyfriend that she does not wish to have sex with him because it is against the law. She might not wish to have sex with him, but she wouldn't want him to laugh at her either.
(c) Focus group discussions with 11–16 year-olds reveal that they are generally opposed to a change in the law on this matter.^9^ This is indeed the case, but, as indicated above, there is a marked disparity between the behaviour of young people and their views on the existing law.
(d) Young people aged 14 years are not physically mature enough to engage in full sexual activity. The median age of menarche in English and Welsh girls born between 1982 and 1986 was 12 years and 11 months.^10^ Thus, the great majority of girls of 14 years are indeed sufficiently physically mature to engage in full sexual activity.
(e) Young people aged 14 years are not cognitively mature enough to evaluate the risks of engaging in sexual activity. There is ample evidence that 14-year-olds are as capable of analysing the risks and benefits of different interventions in complex medical situations as are 21-year-olds.^11^
(f) Young people aged 14 years are not emotionally mature enough to engage in full sexual activity. Steinberg^12^ – while accepting that mid-teenagers have sufficient cognitive maturity – suggests there is evidence that this is not the case when they are emotionally aroused or exposed to peer pressure. In particular, he cites his own work^13^ pointing to age differences in sensation-seeking and impulsivity. These studies of young people aged from 10 to 11 years up to 30 years show reduction of impulsivity with increasing age under experimental conditions. The problem with his argument is that the greatest reduction in impulsivity occurs between adults aged 22–25 and those aged 26–30 years. Is it really suggested that sexual consent should be invalid up to the age of 26 years?
(g) Neuroscientific evidence suggests that the adolescent brain undergoes significant changes throughout the teens and beyond. For example, Casey *et al*^14^ report that ‘recent human imaging and animal studies provide a biological basis […] suggesting differential development of subcortical limbic systems relative to top-down control systems during adolescence relative to childhood and adulthood’. This is taken to mean that those in their teens are not physiologically competent to make important decisions relating to risk-taking. It is surely unwise to rely on such indirect evidence when much more directly relevant studies suggest that it is the inexperience of the young rather than biological limitations that lead to their greater vulnerability in risky situations. For example, McCartt *et al*,^15^ studying traffic accidents among young people, found that ‘of the studies that attempted to quantify the relative importance of age and experience factors, most found a more powerful effect from length of licensure’.

## The arguments in favour

Having effectively countered the arguments against lowering the age of sexual consent, it only remains for me to point briefly to the obvious advantages of such a change in the law.

(a) Lowering the age of sexual consent would result in the decriminalisation of just under one-third of the adolescent population. Most such law-breakers are not currently prosecuted, but it cannot be right that their freely given sexual consent is deemed illegal.
(b) The numbers of young people whose sexual activity results in sexually transmitted infections is substantial.^16^ The number of pregnancies in 15–17-year-olds, although it is reducing, remains substantial.^17^ Further, the sexual experience of many young people, particularly girls, is distressing, and a substantial number of girls regret their first full sexual experience.^18^ Lowering the age of sexual consent would make it distinctly easier for appropriate sex education to be provided to children and young people to enable them to make wiser decisions. It would also make it easier to provide sexual health services to people of this age without the fear of conniving in illegal activity.

Note that it is not proposed here that there should be any changes in the position of those adults who abuse their positions of trust to have sex with people younger than themselves. Further, it is firmly accepted there should be a minimum age limit for sexual consent, a view that has been contested. It is important that it remains recognised that children under the age of 14 years have neither the cognitive nor the emotional maturity to make decisions about their own sexual behaviour.

It will not have escaped the notice of the attentive reader that the principles and evidence adduced here are also relevant to a number of other situations in which the current legal position of minors is highly questionable. For example, at the present time, the age of criminal responsibility in England and Wales is 10 years, while in Scotland it is currently 8 years, with 12 years as the age of criminal prosecution. The age of criminal responsibility should surely be raised to 14 years. The voting age in England and Wales is currently 18 years, while in Scotland it is 16 years. The voting age should surely be reduced to 16 years in England and Wales, with an expectation of a further reduction in due course.

## About the author

**Philip Graham** is Emeritus Professor of Child Psychiatry at the Institute of Child Health, University College London, UK.

## References

[1] Cohen T. Prime Minister swiftly rejects top health experts call to reduce age of consent to 15. *MailOnline*; 18 November 2013.

[2] Family Planning Association. Fact Sheet: The Law on Sex. FPA, 2015.

[3] Home Office. Children and Families: Safer from Sexual Crime – The Sexual Offences Act 2003. Home Office Communications Directorate, 2004.

[4] Guangxing Z, van der Aa S. Trends of age of consent legislation in Europe: a comparative study of 59 jurisdictions on the European continent. N J Eur Crim Law 2017; 8: 14–42.

[5] Graupner H. Sexual consent: the criminal law in Europe and overseas. Arch Sex Behav 2000; 29: 415–61. 10983249 10.1023/a:1001986103125

[6] Waites M. The Age of Consent: Young People, Sexuality and Citizenship. Palgrave Macmillan, 2005.

[7] Mercer C, Tanton C, Prah P, Erens B, Sonnenberg P, Clifton S, Changes in sexual attitudes and lifestyles in Britain through the life course and over time: findings from the National Surveys of Sexual Attitudes and Lifestyles. Lancet 2013; 382(9907): 1781–94. 24286784 10.1016/S0140-6736(13)62035-8 PMC3899021

[8] Loewensen P, Ireland M, Resnick M. Primary and secondary sexual abstinence in high school students. J Adolesc Health 2004; 34: 209–15. 14967344 10.1016/j.jadohealth.2003.05.002

[9] Thomson R. ‘An adult thing’: young people's perspectives on the heterosexual age of consent. Sexualities 2004; 7: 133–49.

[10] Whincup P, Gilg J, Odoki K, Taylor S, Cook D. Age of menarche in contemporary British teenagers: survey of girls born between 1982 and 1986. BMJ 2001; 322: 1095. 11337438 10.1136/bmj.322.7294.1095 PMC31261

[11] Weithorn L, Campbell S. The competency of children to make informed treatment decisions. Child Dev 1982; 53: 1589–98. 7172783

[12] Steinberg L. Does recent research on adolescent brain development inform the mature minor doctrine? J Med Philos 2013; 38: 256–67. 23607975 10.1093/jmp/jht017

[13] Steinberg L, Albert D, Cauffman E, Banich M, Graham S, Woolard J. Age differences in sensation seeking and impulsivity as indexed by behavior and self-report: evidence for a dual systems model. Dev Psychol 2008; 44: 1764–8. 18999337 10.1037/a0012955

[14] Casey B, Jones R, Somerville L. Braking and accelerating of the adolescent brain. J Res Adolesc 2011; 21: 21–33. 21475613 10.1111/j.1532-7795.2010.00712.x PMC3070306

[15] McCartt A, Mayhew D, Braitman K, Ferguson S, Simpson H. Effects of age and experience on young driver crashes: review of recent literature. Traffic Inj Prev 2009; 10: 209–19. 19452361 10.1080/15389580802677807

[16] Public Health England. Sexually transmitted infections and chlamydia screening, 2016. Health Prot Rep 2017; 11(20).

[17] Office for National Statistics. Conceptions in England and Wales: 2014. ONS, 2016.

[18] Wight D, Henderson M, Raab G, Abraham M, Buston K, Scott S, Hart G. Extent of regretted sexual intercourse among teenagers in Scotland: a cross-sectional survey. BMJ 2000; 320: 1243. 10797033 10.1136/bmj.320.7244.1243 PMC27366

